# High-Sensitivity in Dielectrophoresis Separations

**DOI:** 10.3390/mi11040391

**Published:** 2020-04-09

**Authors:** Benjamin G. Hawkins, Nelson Lai, David S. Clague

**Affiliations:** Biomedical Engineering Department, California Polytechnic State University, San Luis Obispo, CA 93407, USA; nelai@calpoly.edu (N.L.); dclague@calpoly.edu (D.S.C.)

**Keywords:** dielectrophoresis, microfluidics, cell separation

## Abstract

The applications of dielectrophoretic (DEP) techniques for the manipulation of cells in a label-free fashion within microfluidic systems continue to grow. However, a limited number of methods exist for making highly sensitive separations that can isolate subtle phenotypic differences within a population of cells. This paper explores efforts to leverage that most compelling aspect of DEP—an actuation force that depends on particle electrical properties—in the background of phenotypic variations in cell size. Several promising approaches, centering around the application of multiple electric fields with spatially mapped magnitude and/or frequencies, are expanding the capability of DEP cell separation.

## 1. Introduction

Dielectrophoresis (DEP) is in the process of growing from a developmental technology to an integrated research tool. A testament to this is the number of research articles that continue to demonstrate novel device designs and separations. There are number of excellent recent review articles that establish a clear foundation in the theory [1], technologies [2,3], and applications [4,5,6]. With many preeminent researchers, an active research portfolio, and strong surveys of the field, there is little need at this time for a broad survey of DEP theory and applications; instead, this review focuses specifically on high-performance techniques that aim to achieve highly sensitive separations and overcome some of the traditional challenges found in DEP applications. For completeness and context, however, a short summary of the foundational physics and assumptions that are integrated in the oft-cited governing equations for DEP are presented here.

DEP is the transport of polarizable particles in response to an externally applied electric field. Actuation is achieved by application of a non-uniform electric field which simultaneously induces polarization and exerts force on the interface between two electrically dissimilar media. In general, the DEP force can be written in terms of Maxwell’s Stress Tensor, $\overset{⇉}{T}$:

(1) $$\begin{array}{c} {\vec{F}}_{DEP}={\;\int \int \int \;}_{V}\left(\nabla \cdot \overset{⇉}{T}\right)dV \end{array}$$

(2) $$\begin{array}{c} \overset{⇉}{T}=\epsilon \vec{E}\vec{E}+\frac{1}{\mu }\vec{B}\vec{B}-\frac{1}{2}\left(\epsilon \vec{E}\cdot \vec{E}+\frac{1}{\mu }\vec{B}\cdot \vec{B}\right)\overset{⇉}{I}, \end{array}$$

where $\vec{E}$ is the electric field, $\vec{B}$ is the magnetic field, $\vec{n}$ is the unit normal to a surface over which we are integrating to calculate the total force, ϵ is the material permittivity, μ is the material permeability, and $\overset{⇉}{I}$ is the identity tensor. Equation (2) is an expression of the volumetric electromagnetic force on an object with non-uniform electrical properties. Though the DEP force can be generated by static or time-varying fields, the applications of interest here—those that achieve some form of separation that is sensitive to particle electrical properties—typically employ harmonic (sinusoidal) electric fields with frequencies that allow a quasi-static approximation. The force observed is the result of time-averaging the DEP force equation [7]:

(3) $$\begin{array}{c} \langle {\vec{F}}_{DEP}\rangle ={\;\int \int \int \;}_{V}\left(\nabla \cdot \langle \overset{⇉}{T}\rangle \right)dV \end{array}$$

(4) $$\begin{array}{c} \langle \overset{⇉}{T}\rangle =\frac{1}{4}ℜ\left(\tilde{\epsilon }\right)\left(\vec{E}{\vec{E}}^{*}+{\vec{E}}^{*}\vec{E}-{\left|\vec{E}\right|}^{2}\overset{⇉}{I}\right), \end{array}$$

where ${\vec{E}}^{*}$ is the complex conjugate of the electric field. An accurate model for the force on a particle would be based on the volume integral of divergences of the MST within the particle. Application of the divergence theorem and assumption of locally homogeneous electrical properties allows replacement of Equation (4) with:

(5) $$\begin{array}{c} \langle {\vec{F}}_{DEP}\rangle ={\;\int \int \;}_{A}\left(\langle \overset{⇉}{T}\rangle \cdot \vec{n}\right)dA, \end{array}$$

where $\vec{n}$ is the unit vector normal to a surface at the interface between two media with homogeneous, but dissimilar, electrical properties and indicates that DEP force is generated at teh interfaces between materials. This simplification provides the basis for the multi-shell models employed by most researchers in the field.

### Approximations

With the application of several approximations, the expression for ${\vec{F}}_{DEP}$ can be simplified to the form presented in most applications in literature:

(6) $$\langle {\vec{F}}_{DEP}\rangle =\pi \epsilon_{m}a^{3}ℜ\left({\tilde{f}}_{CM}\right)\nabla \left(\vec{E}\cdot \vec{E}\right),$$

where ${\tilde{f}}_{CM}$ is the complex Clausius-Mossotti factor and *a* is the particle radius. ${\tilde{f}}_{CM}$ depends on the complex permittivity, $\tilde{\epsilon }$ of material on either side of the interface under consideration:

(7) $$\begin{array}{c} {\tilde{f}}_{CM}=\frac{{\tilde{\epsilon }}_{p}-{\tilde{\epsilon }}_{m}}{{\tilde{\epsilon }}_{p}+2{\tilde{\epsilon }}_{m}} \end{array}$$

(8) $$\begin{array}{c} \tilde{\epsilon }=\epsilon -j\frac{\sigma }{\omega }, \end{array}$$

where ϵ is the electrical permittivity, σ is the electrical conductivity, and ω is the frequency of the applied electric field in radians/second. $ℜ({\tilde{f}}_{CM})$ varies from −0.5 to 1.0; the point where $ℜ({\tilde{f}}_{CM})=0$ is termed the “cross-over frequency” and here notated as $f_{CM,0}$. The approximations that lead to Equation (6) should be carefully examined for a given application:

- **Isotropic Media** The material on either side of the interface is assumed to have electrical properties that are independent of the orientation of the electric field.
- **Homogeneous Media** The material on either side of the interface is assumed to have spatially uniform electrical properties.
- **Spherical Particle** The most common assumption addressed is the spherical particle assumption. Good approximations exist for spheroidal particles and are often employed when particle shape deviates significantly from spherical.
- **Semi-infinite Domain** The domain is assumed to be large relative to the size of the particle; this also assumes that the particle is not in close proximity to other particles. Other particles perturb the local electric field solution and alter the DEP force.
- **Dipole Field** Equation (6) assumes that the perturbation to the externally applied, non-linear electric field is well-approximated by an equivalent dipole, that is, that the variation of the field over the particle is approximately linear. Multipole terms exist and can be used for suitably non-linear electric field gradients [8].

## 2. Sensitivity

Here, we consider the effects of variability among the major components of a multi-shell model for a subjected to a DEP force. To examine the sensitivity of the magnitude of the DEP force to changes in cellular components, we consider the magnitude of the DEP force normalized by the squared field gradient, which we define as $\Gamma_{DEP}$:

(9) $$\begin{array}{c} \Gamma_{DEP}\equiv \left(\frac{{\vec{F}}_{DEP}}{\nabla (\vec{E}\cdot \vec{E})}\right)=\pi a^{3}\varepsilon_{m}ℜ\left({\tilde{f}}_{CM}\right). \end{array}$$

We consider a 4-shell, spherical cell with parameters reported by Rohani, et al. [9] and indicated in Table 1. This work is further discussed in Section 3.5. This model includes permittivity and conductivity for the cell membrane, cytoplasm, nuclear envelope, and nucleoplasm. While no definitive library exists for such parameters, a survey of available literature indicates that permittivity values vary slightly compared to conductivity values for each of these cellular compartments.

**Figure 1:**
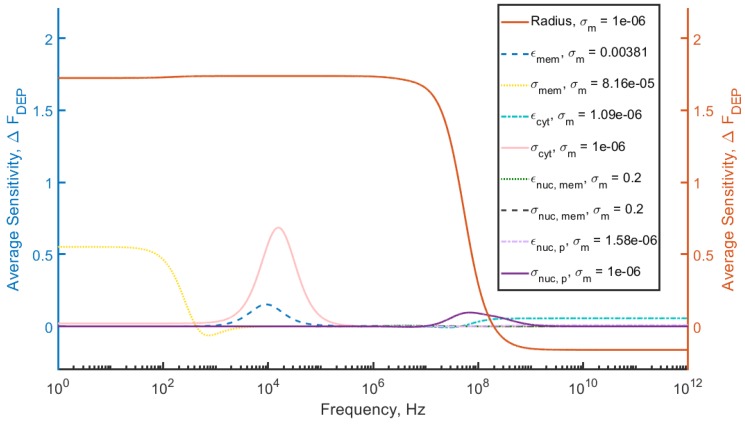
The average variation in the DEP force, $\Delta F_{DEP}$, that arises from variations in radius and other electrical parameters in a 4-shell model for HEK cells. Model parameters are given in Table 1.

To determine the sensitivity, a MATLAB model was developed to calculate $\Gamma_{DEP}$ between 1 Hz and $10^{12}$ Hz for a given set of electrical model cell parameters. Values for $\Gamma_{DEP}$ were calculated for a 10% increase of permittivity and a 100% increase of conductivity for each compartment over 10 equally spaced intervals, for example, Δε or Δσ. The average of these changes at each frequency was calculated to determine the sensitivity as a function of frequency for each parameter.

(10) $$\begin{array}{c} \Delta F_{DEP}=\frac{1}{N-1}\sum_{j=1}^{N}\left|\Gamma_{DEP,j}-\Gamma_{DEP,j+1}\right|, \end{array}$$

with summation over *j* representing the interval. The multishell model equation is a monotonic function for the values considered, so refinement of the intervals would yield no additional information. As shown by Rohani, et al. and others [10], media conductivity can be chosen to enhance the variation of the DEP force in response to changes in cell properties. Accordingly, MATLAB’s optimization package was used to vary media conductivity between 1μS/m and 0.2 S/m to maximize the variation of $\Delta F_{DEP}$ in response to changes in a given compartment electrical parameter, independent of frequency. The optimized conductivities for each parameter are indicated in Table 2 and Figure 1.

The average sensitivity of the DEP force, $\Delta F_{DEP}$, is plotted as a function of frequency in Figure 1.

**Figure 2:**
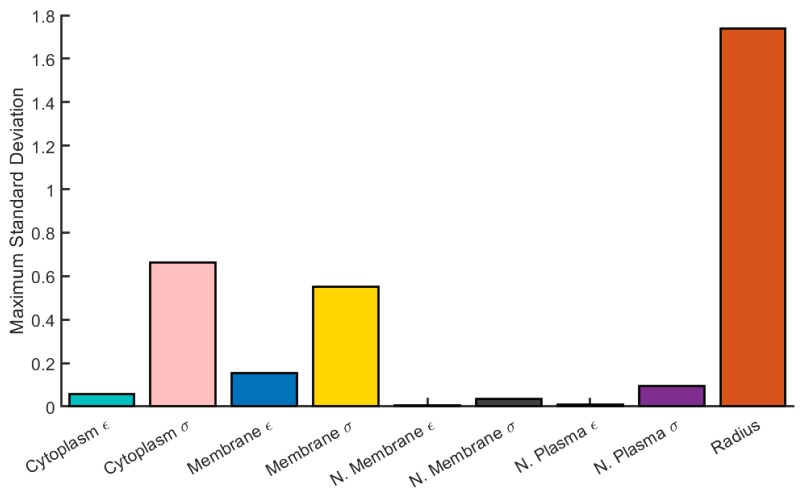
The maximum average variation in $\Delta F_{DEP}$ across all frequencies that arises from variations in radius and other electrical parameters in a 4-shell model for HEK cells. Model parameters are given in Table 1.

The largest change in the DEP force is due to variation in cell radius, with other parameters contributing significantly less (by approximately a factor of 2) to the overall variation. The maximum average variation across all frequencies was considered as well (Figure 2) and shows similar results.

In order to achieve the optimized sensitivity, solutions of quite high or low conductivity are required, potentially harming or altering cell populations of interest, so the realistic limits for the relative contributions of cellular electrical properties to the overall DEP force remains significantly lower than that of cell size. This underscores the need for DEP techniques that aim to reduce the contribution of overall size variation.

**Figure 3:**
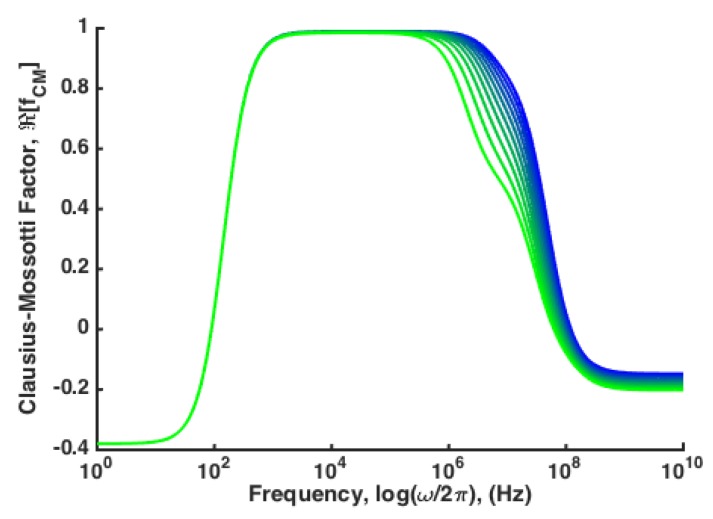
A multi-shell model that incorporates the presence of increasing volume fraction of Hepatitus-C viral vesicles in an example ellipsoidal hepatocyte model. The primary variation that results is a change in the magnitude of pDEP at high frequencies.

Beyond the need for sensitive techniques that can reduce the influence of biological size variability, DEP techniques are needed that can distinguish between subtle variations in the magnitude of the force, rather than the cross-over frequency. For example, a model for viral vesicle inclusions in Hepatitis C infected hepatocytes is developed using a combination of multi-shell models and Maxwell’s mixture theorem. As Hepatitis-C virus infection continues, vesicles containing viral bodies build up within the cytoplasm of hepatocytes. Isolation of these cells from a background of healthy hepatocytes could have significant impact on diagnosis and treatment. The results indicate that increasing viral vesicle load within the cytoplasm causes subtle variation in $ℜ({\tilde{f}}_{CM})$ in the pDEP frequency regime and relatively small variation in the cross-over frequency, $f_{CM,0}$ (Figure 3) [11].

In order to realize the potential of DEP to perform truly robust, repeatable, label-free separations, a collection of techniques and approaches must be developed that can successfully separate cells with subtle variations like those identified in Figure 3 independent of biological size variability.

## 3. Approaches to Increasing Sensitivity

In general, methods to improve the sensitivity of DEP separations introduce another force that acts in opposition to the primary actuating DEP force and also carries with it a size-dependence. In this way, if the opposing force is volume-dependent, the effects of particle size can largely be omitted. Here we consider the primary methods for generating an opposing force and characterize each method by whether it is a batch process or continuous flow, whether it is an equilibrium method or not, and whether it is a dynamic process or static. For clarity, batch processes are those that separate a population of particles once, and must be cleaned and reloaded to perform additional separation; continuous flow separations operate continuously and do not require “resetting”. Equilibrium methods separate particles to a particular position where the DEP force is cancelled out by the opposing force; non-equilibrium methods have a net force on actuated particles, so they will continue to move in response. Dynamic separations are those that change the separation or actuation force during the separation process, static separations yield a single result for the separation of particles. The highest performance DEP separation will be one that achieves continuous, equilibrium separation in a dynamically adjustable manner.

### 3.1. Gravity-Based Systems: DEP-FFF

The first practical method to counteract the size-dependence of DEP was demonstrated by Wang, et al. [12], and used gravity to perform a combination of DEP and field flow fractionation (FFF). Subsequently, the process has been refined and separations of differentiated stem cells, [13], circulating tumor cells [14,15] and bacteria [16] have been demonstrated.

In a recent ‘tour-de-force’ in the application of DEP-FFF, Gascoyne and co-workers [14,17,18] demonstrated isolation of circulating tumor cells (CTCs) from blood, specifically preferentially capturing CTCs over peripheral blood mononuclear cells. This demonstration, overcoming one of the main confounding factors that reduce the purity of other separation methods, highlights the need for further development of DEP technologies. The DEP-FFF method used for capture and characterization in this suite of studies was based on the elution time in a DEP-FFF system; operating as a batch, non-equilibrium, dynamic method. In addition to CTC isolation and characterization, Waheed, et al. identified a “dielectric phenotype” factor (1/Rϕ) that depends on the particle radius (*R*) and the degree of membrane folding (ϕ). This factor was shown to be the primary determining factor of the cell cross-over frequency, $f_{CM,0}$ [14].

DEP-FFF suffers from a number of confounding factors, however, that limit its size-independent nature. The reliance on a pressure-driven flow field that varies significantly over the size of the typical particle leads to a drag induced torque and subsequent rotational lift force in certain flow regimes [19]. In addition to this lift effect, DEP-FFF methods lead to a equilibrium separation position that is a function of device depth, so orientation of the device to gravity is a factor and recovering separate streams from such a separation poses very challenging fabrication [20]. Separating multiple streams vertically within a microfabricated system requires multilayer device alignment, increasing design and manufacturing cost. For this reason, DEP-FFF is typically operated as a batch process, with elution time being the primary measurement.

### 3.2. 2D Electrode Systems: Single-Field/Single-Frequency

There are several methods that utilize DEP trapping effects to reduce the size-dependent nature of the force. In contrast to DEP-FFF, where the DEP force and fluid drag forces are orthogonal, these techniques typically involves a balance between nDEP forces and fluid drag in the same direction which reduces—but does not eliminate—the radius term. When these forces are balanced, the resulting DEP velocity is dependent on $a^{2}$,

(11) $$\begin{array}{c} {\vec{u}}_{DEP}=\frac{\varepsilon_{m}a^{2}ℜ\left({\tilde{f}}_{CM}\right)}{6\eta }\nabla \left(\vec{E}\cdot \vec{E}\right), \end{array}$$

where η is the fluid viscosity. As a result, the approach to achieving size-independent separations using a single DEP force relies on leveraging the so-called “cross-over frequency” of DEP force. In this manner, a single field with a single frequency can be applied to a device, and particles experiencing a non-zero DEP force will be actuated, with those at the cross over frequency being unaffected. This approach has been used to separate cells from latex beads [21], bacteria from blood [22], and fluorescently-labeled cancer cells from blood [4]. Electrode configurations that are employed consist of interdigitated [23,24], castellated [25], ratchet [26] and trapezoidal [27] configurations.

There are a few limitations to this approach—(i) the dependence on the cross over frequency, (ii) the near-zero value of the DEP force near the cross over frequency, and (iii) the binary nature of the separation mechanism. In consideration of the former, many alterations to the electrical properties of particles of interest do lead to changes in the cross-over frequency, however there are many examples of more subtle changes that could be actuated via DEP that do not lead to significant changes in cross-over frequency (see Figure 3). While the force at cross over is zero, and therefore the lack of actuation from DEP will be independent of size, the magnitude of the DEP force near the cross over frequency is small, and other size-dependent actuation forces will be potentially dominant for particles with $ℜ({\tilde{f}}_{CM})$ near the cross over frequency, again leading to a reduction in sensitivity and an increase in size dependence. Finally, the binary nature of this approach leads to a separation for particles that have a cross over frequency near the field frequency, and those that do not. There is no ability to separate a spectrum of particles as a function of cross-over frequency. These factors combined mean that single-field, single-frequency approaches lack sufficient resolving power to truly engage the potential of DEP for highly sensitive separations.

The concept of “iso-motive” DEP also rests within this category, with electrode configurations that generate a spatially uniform DEP force (hence “iso-motive”) [28,29]. The approach counters one of the challenges to DEP—the non-uniform nature of the force—but all the above approaches lead to batch-mode separations, or continuous-flow separations that remain size-dependent.

Of particular interest for this review is the application of CMOS memory fabrication techniques to realize an addressable electrode grid in a microchannel [30]. The authors created an array of 32,768 individually addressable 11μm-square electrodes. Bordering on hyperresolution digital microfluidics, the ability to apply an RF-frequency field to each local electrode leads to exceptionally promising platform for highly flexible separation design and testing. In contrast to many purpose-built DEP separation systems, this programmable array integrated circuit approach could be dynamically reconfigured to a wide range of separations, or tuned for a specific separation in-situ.

### 3.3. 2D Electrode Systems: Multiple-Fields/Multiple-Frequencies

The combination of multiple electric fields operating at the same or different frequencies holds the greatest promise for highly sensitive DEP separations. Multiple-field configurations can be split in to two major types: combined field DEP and travelling-wave DEP. Multiple, overlapping fields have been generated in planar electrode configurations and leveraged by many to accomplish various cell separations. In the combined-field separation area, work by Urdaneta and Smela showed separation of live and dead yeast cells, using different frequencies to preferentially attract each cell type to a different set of electrodes [31]. Similar techniques have been recently applied, taking advantage of multi-frequency signals (amplitude, frequency, or phase modulated) on a single electrode array to generate dissimilar DEP actuation forces on particles of interest to separate polystyrene microspheres based on size [32], algae cells based on lipid content [33], and MCF7 cancer cells from diluted blood [34]. Planar multifield configurations have also been extended to concentrate viruses, proteins, and bacteria [35].

In a thoughtful extension of DEP-FFF, Gascoyne and coworkers developed the “electrosmear” assay by spatially mapping the magnitude of the DEP force along the channel [36]. nDEP forces are generated on the bottom of a wide channel, and variations in the magnitude of the DEP force are accomplished by changing the local electric field magnitude or by changing the local electric field frequency. This, combined with the gravitational sedimentation force leads to cell adhesion to the interdigitated electrode array at locations where the nDEP force is overcome by gravity. This allows a spatial mapping of cellular DEP response to physical location on the electrode array. The electrosmear technique effectively minimizes size dependence, but because it is a temporal (batch) separation technique, it is relatively low throughput. Also, as with DEP-FFF, dependence on gravity limits the flexibility and versatility of the system.

Travelling-wave approaches leverage an electric field that is typically applied across 4 electrodes, each $90^{\circ }$ out of phase with one another, leading to a field force that is based on the out of phase component of ${\tilde{f}}_{CM}$ [37,38]:

(12) $$\begin{array}{c} \langle {\vec{F}}_{twDEP}\rangle =-\frac{2\pi^{2}a^{2}\varepsilon_{m}}{\lambda }ℑ\left({\tilde{f}}_{CM}\right)\nabla \left(\vec{E}\cdot \vec{E}\right), \end{array}$$

where $ℑ\left(\cdots \right)$ refers to the imaginary part and λ defines the wavelength of the twDEP field (i.e., distance between electrodes at each phase). twDEP systems have the advantage of reduced size dependence and can be applied perpendicular to the drag force and the electrode array, meaning that the twDEP field simultaneoulsy suspends and translates cells. The electrodes are actuated at a frequency corresponding to nDEP, suspending them above the array, and the difference in phase drives them transverse to the array. One of the initial applications was developed in theory by the Mezic group to demonstrate a multifield appraoch using twDEP to achieve separation [39]. twDEP methods have also been used by many and show promise as a method for size-independent cell separation [40,41,42,43]. In each case, the twDEP field is used to simultaneously levitate cells via nDEP and transport them transverse to the direction of a fluid flow field via the phase-dependent component of the twDEP force (proportional to $ℑ({\tilde{f}}_{CM}$). However, twDEP remains a non-equilibrium technique. In order to achieve predictable separation, careful tuning of force magnitude and flow velocity is required, or binary separation can be achieved as the equilibrium condition.

### 3.4. 3D Electrode Systems

Three-dimensional electrode systems refers to the use of electrodes on multiple sides of a microfluidic channel, rather than confining the electrode pattern to one side. This can mean an energized channel top with an interdigitated array on the bottom, interdigitated arrays embedded in the side of a microchannel, conductive posts, or electrodes that encompass the entire channel. Some 3D electrode systems also have incorporated multiple electric fields at different frequencies.

In a straightforward extension of the planar interdigitated electrode array, Lai, et al. used a solid conductive ITO sheet to close the top of the channel and energized an interdigitated array formed using a Ti/Al thin film on the bottom. The device successfully isolated RBCs from plasma in a serpentine channel [25]. Another example of top/bottom electrode configuration for continuous flow was demonstrated by Tada, et al. using multiple fields applied to an interdigitated array on the channel bottom with an indium-tin-oxide (ITO) coated glass top layer to generate electric field gradients spanning channel depth. The result was a device that trapped dead cells via pDEP on the channel bottom and concentrated live cells via opposing nDEP forces in the center of the channel [44]. Top-bottom electrode configurations generate gradients throughout channel depth, overcoming the challenge faced by planar configurations whose gradients do not reach sufficiently far in to the device to affect all particles. The distribution of field gradients allows for better control over their location as well, but at the cost of lower gradient magnitudes, meaning such devices require larger applied fields to achieve actuation. While top/bottom configurations offer better uniformity across channel depth, they require precise alignment if electrodes are patterned on both sides of the device, or suffer slightly non-uniform gradients across channel depth if one surface is a single electrode. Separations that leverage the top/bottom electrode configuration either rely on the cumulative effects of successive particle-gradient interactions or repulsion type interactions to manipulate particles. Applying a single field between electrodes leads to separations that depend on the equilibrium between nDEP and fluid drag forces, as shown in Equation (11). While features can be patterned to achieve better control over particle trajectories, making for more sensitive separations that can be performed in continuous flow, they remain strongly size-dependent.

Spanning the field from top to bottom of the channel has been accomplished with physical structures as well. Energized post array systems have been developed by Voldman and co-workers to achieve broad field distributions that were able to achieve single-cell trapping [45]. These traps were extruded metal posts that generated a quadrupolar DEP force field. Martinez-Duarte and co-workers have demonstrated a technique to easily generate arrays of conductive pillars by pyrolysis of SU-8 and used the resulting structures to filter bacterial cells [46,47]. This approach is highly efficient for fabrication and allows distribution of the electric field throughout the depth of the microchannel. These structures overcome the challenge of varying field magnitude across channel depth encountered in the top/bottom configurations. Conductive pillars allow control of field gradients uniformly across channel depth without requiring precise alignment of top and bottom structures, and require lower field magnitudes. Like top/bottom electrode configurations, applying a single field leads to continuous-flow separations that rely on successive interactions or exclusion, and remain size dependent.

Electrodes have also been incorporated in to the sidewall of a channel, fashioned out of conductive PDMS [48], solder [49], and various metals [50,51]. The advantage of 3D- and sidewall electrode systems is that they distribute the electric field (and its gradient) throughout the channel. Rather than limited by proximity to a planar interdigitated electrode array on a single channel surface, placing potential and ground on opposite sides of the channel allows the DEP force to be applied further in to the fluid domain. Extending the application, electrodes on either sidewall have been developed to achieve lateral separation of a number of analytes [14,52,53]. By changing the distance between electrodes the field magnitude can be spatially mapped, and cell separation can be tuned even further, improving sensitivity and allowing for either batch or continuous separations. Without a countering, volume dependent force, however, the resulting separation is size-dependent.

A few researchers have demonstrated successful actuation of cells using 3D electrode structures and multiple field frequencies, successfully sorting particles transverse to the direction of flow and achieving a continuous flow, size-independent separation [54]. In this work, opposing nDEP and pDEP fields were applied to side-wall electrodes to separate HEK293 from N115 mouse neuroblastoma cells. The benefits of such a system are clear. The total DEP force exerted on a particle due to overlapping fields at different frequencies. Under ideal circumstances, the field applied at the sidewall exerts a force transverse to the direction of flow such that the equilibrium position of a single particle can be determined according to:

$$\begin{array}{c} \sum {\vec{F}}_{DEP}=2\pi \varepsilon_{m}a^{3}ℜ\left({\tilde{f}}_{CM,1}\right)\nabla \left({\vec{E}}_{rms,1}\cdot {\vec{E}}_{rms,1}\right)+ \end{array}$$

(13) $$\begin{array}{c} 2\pi \varepsilon_{m}a^{3}ℜ\left({\tilde{f}}_{CM,2}\right)\nabla \left({\vec{E}}_{rms,2}\cdot {\vec{E}}_{rms,2}\right)=0 \end{array}$$

(14) $$\begin{array}{c} \frac{\nabla \left({\vec{E}}_{rms,1}\cdot {\vec{E}}_{rms,1}\right)}{\nabla \left({\vec{E}}_{rms,2}\cdot {\vec{E}}_{rms,2}\right)}=\frac{ℜ\left({\tilde{f}}_{CM,2}\right)}{ℜ\left({\tilde{f}}_{CM,1}\right)}, \end{array}$$

where the equilibrium lateral position of a particle subjected to fields with different frequency and magnitude occurs at the point where the ratio of squared field gradients equals the ratio of Clausius-Mossotti factors. This approach indicates four possible experimental tuning parameters—field magnitudes and field frequencies. While this approach achieves size-independence, application of the field across the channel width requires high field magnitudes or narrow channel dimensions. Furthermore, gradients between electrodes decay rapidly, meaning that a component of the applied DEP forces oppose fluid drag and potentially reintroduce a measure of size-dependence.

The furthest extension of the 3D electrode based dielectrophoresis concept was demonstrated by Kung, et al. and termed “tunnel” DEP. Researchers fabricated independent electrodes at the corners of a microchannel parallel to the direction of flow. Selective actuation of the electrodes at each corner allowed positioning of a stream of particles to an equilibrium position within the channel cross-section [55,56]. Like Wang, et al. this size-independent separation offers advantages in the form of tunability and continuous flow separation; additionally, this approach offers a second dimension of spatial control, in theory allowing the positioning of particles within the channel cross section based on DEP responses to multiple field frequencies and magnitudes. In order to achieve this level of control, however, complicated fabrication procedures must be applied and channel dimension must remain small to maintain field gradient magnitudes.

### 3.5. Media Conductivity

In addition to cell electrical properties, the DEP force depends on the properties of the surrounding media. Throughout it development, researchers have chosen media conductivity to allow for larger DEP force magnitudes and potential separations. Swami and co-workers have demonstrated a number of sensitive separations, isolating cells with mitochondrial structure variations [9], extracellular vesicles from pancreatic tumor cells based on invasiveness [57], and bacterial (Clostridium difficile) cells with altered envelope structure [58]. While the approach does not eliminate size dependence, as we show in Section 2, it can be leveraged to increase the relative contribution of particle electrical properties to variations in the resultant DEP force.

In another approach to minimize the dependence on size in cell discrimination and increase sensitivity, Voldman and coworkers developed “isodielectric focusing” which leverages the media dependence of ${\tilde{f}}_{CM}$ eliminating the majority of size dependence in a continuous flow separation [59,60]. Vahey, et al. established the isodielectric focusing technique by first creating a spatial gradient in the electrical conductivity of the media. Suspended cells then have a varying ${\tilde{f}}_{CM}$ across the gradient. Using an angled array of interdigitated electrodes to induce an nDEP force, they were able to deflect polystyrene beads and cells to a location where the nDEP force goes to 0. Particles introduced by a pressure driven flow are deflected transverse to the direction of flow—and transverse to the media conductivity gradient—by nDEP. As the local media conductivity changes, ${\tilde{f}}_{CM}$ changes and approaches 0. When ${\tilde{f}}_{CM}=0$, $\langle {\vec{F}}_{DEP}\rangle =0$ and particles are transported by fluid flow. While this technique also minimizes size dependence and achieves continuous flow separation, it requires a conductivity gradient that is both stable and properly varies $ℜ\left({\tilde{f}}_{CM}\right)$ to achieve separation. Such a stable flow and proper design of the conductivity gradient may not be achievable in all applications.

## 4. Conclusions

In this article, we have identified a number of approaches that variously combine 2D and 3D electrode structures, single or multiple electric field magnitudes, single or multiple electric field frequencies, and media variation strategies aimed at improving the sensitivity of label-free DEP separations to small variations in cell electrical properties. An analysis of the sensitivity of the DEP force indicates that particle size variation is potentially the largest factor, confounding efforts to isolate other variations. Therefore, we focus on efforts to increase sensitivity while also reducing or eliminating size dependence. The first counteracting force was gravity. DEP-FFF encompases these techniques and there is a deep body of work in the area. The technique has the potential to be sensitive and size-independent, but either operates in batch mode, limiting throughput, or requires significant manufacturing investment to develop a continuous flow implementation. 2D electrodes are often used to actuate DEP forces. For the most part, these techniques can be sensitive, and are easy to fabricate, but without an opposing force, are largely size dependent. 2D electrode structures where multiple field magnitudes and/or multiple field frequencies are applied overcome the size-dependent nature with a second DEP force. While eliminating the size-dependence, 2D structures offer limited control of the spatial distribution of electric field gradients. 3D electrode structures, on the other hand, distribute electric field gradients more uniformly, at the expense of slightly more involved fabrication, but without multiple fields, remain size-dependent. Combining 3D structures with multiple fields overcomes both the size-dependent nature of the DEP force as well as the challenge of controlling the spatial distribution of the electric field.

As we develop techniques that get closer and closer to isolating minute particle electrical properties, it is highly likely that our assumptions of homogeneity and isotropy will break down. The multi-shell model, in these cases, will likely not completely reflect or predict particle response. In order to more accurately model the DEP force, some have considered multipolar approximations, but the MST approach (Equation (2)), should be considered when attempting to model particle behavior and characterizing these highly sensitive separations.

## 5. Future Directions

The field of DEP cell separation continues to iterate on new device designs and configurations that take advantage of various chemical, hydrodynamic, and electrical properties of devices and analytes. The field is bustling with new proof of concept demonstrations that achieve challenging separations in useful ways. As microfabrication techniques become more and more accessible and see application to the microfluidic field in more useful ways, dielectrophoresis and electrokinetics will play a crucial role in new and novel separations that answer valuable research questions.

The biggest challenge to the field will be successfully bridging the gap between increasingly niche and narrowly focused demonstrations of a particular separation to robust and flexible separation tools that can be routinely applied in the biomedical and basic science labs. To date, DEP has not successfully bridged that gap, with DEP-FFF and the DEPTech 3DEP instrument being notable exceptions. The potential for truly label-free separations based solely on subtle variations in electrical phenotype in a robust, repeatable, and flexible platform remains a persistent challenge. Novel approaches that leverage spatially tunable electric field magnitudes and frequencies will be at the forefront of the next generation of DEP separation devices; tools and instruments that will enable groundbreaking work in the hands of a broader community of biomedical, biological, and basic science researchers.

## Figures and Tables

**Table 1:** Model parameters for DEP force sensitivity evaluation. These baseline values were used as a starting point and increased by 10% for permittivities and 100% for conductivities. Variation of cell radius increased the overall cell size, but did not change the thickness of any layer.

Variable	Value	Units	Definition
*a*	10.01	μm	Outer cell radius
$a_{m}$	10	μm	Inner membrane radius
$a_{nm}$	5.02	μm	Nuclear outer radius
$a_{np}$	20	nm	Nucleoplasm (inner) radius
$\varepsilon_{mem}$	14	(rel)	Membrane permittivity
$\sigma_{mem}$	0.11	μS/m	Membrane conductivity
$\varepsilon_{cyt}$	60	(rel)	Cytoplasm permittivity
$\sigma_{cyt}$	0.52	S/m	Cytoplasm conductivity
$\varepsilon_{nm}$	25	(rel)	Nucleus membrane permittivity
$\sigma_{nm}$	3	mS/m	Nucleus membrane conductivity
$\varepsilon_{np}$	60	(rel)	Nucleoplasm permittivity
$\sigma_{np}$	1.4	S/m	Nucleoplasm conductivity
$\varepsilon_{m}$	80	(rel)	Media permittivity
$\sigma_{m}$	varies	S/m	Media conductivity

**Table 2:** Optimized media conductivity values to maximize the variation in $\Delta F_{DEP}$ in response to changes in cell model electrical properties.

Variable	Optim. σ Value	Units	Definition
*a*	1	μS/m	Outer cell radius
$\varepsilon_{mem}$	3.81	mS/m	Membrane permittivity
$\sigma_{mem}$	81.6	μS/m	Membrane conductivity
$\varepsilon_{cyt}$	1.09	μS/m	Cytoplasm permittivity
$\sigma_{cyt}$	1	μS/m	Cytoplasm conductivity
$\varepsilon_{nm}$	0.2	S/m	Nucleus membrane permittivity
$\sigma_{nm}$	0.2	S/m	Nucleus membrane conductivity
$\varepsilon_{np}$	1.58	μS/m	Nucleoplasm permittivity
$\sigma_{np}$	1	μS/m	Nucleoplasm conductivity

## Abbreviations

The following abbreviations are used in this manuscript:

MDPI	Multidisciplinary Digital Publishing Institute
DEP	Dielectrophoresis
nDEP	Negative Dielectrophoresis
pDEP	Positive Dielectrophoresis
DEP-FFF	Dielectrophoretic Field-Flow Fractionation
CTC	Circulating Tumor Cell
